# New Books and New Editions

**Published:** 1892-10-15

**Authors:** 


### NEW BOOKS AND NEW EDITIONS.

### SEA SICKNESS.*

This little book, like others published by Dr. Dutton, contains much good advice and sound common sense. Dr. Dutton has no novel theories to advocate as to the causation and pathology of sea sickness, but he gives with simplicity the results of his own experience and method of treatment. He believes that much of the sea sickness endured is caused by the worry, fatigue, and excitement, and the farewell dinners incidental to starting on a long sea journey, so that travellers come on board with deranged stomachs and digestion, which the oscillatory movements of the ship aggravate. He therefore advises that in cases of obstinate sea sickness, of which the patient knows by past experience he will be a victim, the treatment should be begun a fortnight before starting. In these cases he gives a pill composed of podophyllin, euonymin, pepsine, and hyoscyamus every night, and a glass of saline water twice a week before breakfast, and he insists upon regular exercise being taken. Three days before going on board a mixture of bromide of ammonium, sal volatile, and chloroform is prescribed and taken. The dose of bromide of ammonium is increased after starting if sickness occur, and is diminished as it passes away. The horizontal position, lying on deck in the fresh air, and the avoidance of the abundant and greasy food of the ship's dietary are recommended, the substitution of bovinine, hard biscuits, and tea is advised in severe cases, and ten grains of malto-pepsine in those in which food is more or less tolerated. If these directions are followed, Dr. Dutton promises that success will be almost always attained in vanquishing the distressing malady of sea sickness. The value of the drugs used as preventive or curative of sea sickness is discussed, but Dr. Dutton considers that bromide of ammonium is the specific *par excellence*. The book contains some slight though useful information about health resorts in England and abroad. A journey to Cape Town is especially recommended for consumption when the disease is not in an inflammatory stage, but Dr. Dutton deprecates warmly sending patients who are hopelessly and seriously ill to foreign countries, away from the comforts of home and the solace of friends. He has a good word to say for English health resorts such as Torquay, Bath, and Droitwich, where the sanitary arrangements and the food supply can be depended upon, which is more than can be said of many of the much-praised health resorts abroad.

* "Sea Sickness (Cause, Treatment, and Prevention). Voyaging for Health and Health Resorts." By Thomas Dutton, M.D. (Kimpton, London, 1892.)

